# mir-182-5p Regulates Cell Growth of Liver Cancer via Targeting RCAN1

**DOI:** 10.1155/2021/6691305

**Published:** 2021-04-17

**Authors:** Jianxing Zheng, Dongyang Wu, Libing Wang, Fengzhi Qu, Daming Cheng, Xiaogang Liu

**Affiliations:** Department of Hepatobiliary Surgery, Tangshan Gongren Hospital, Tangshan, China

## Abstract

Regulator of calcineurin 1 (RCAN1) is an endogenous protein that is involved in the regulation of the occurrence and progression of a variety of cancers, but currently, people know little about its potential mechanism. This study investigated the function and mechanism of RCAN1 and miR-182-5p in liver cancer cells. In this study, reliable data demonstrated that RCAN1 suppressed cell proliferation, migration, invasion, and cell cycle progression of liver cancer. Additionally, the expression of RCAN1 was noted to be regulated by its upstream regulator miR-182-5p, and miR-182-5p was prominently highly expressed in liver cancer cells. Based on this, it was further proved through cell experiments that miR-182-5p facilitated cell growth of liver cancer through RCAN1 downregulation, showing that RCAN1 may be a fresh biomarker and target for diagnosis and treatment of liver cancer.

## 1. Introduction

According to Cancer Statistics, 2020 [1], liver cancer is the second leading cause of cancer-related deaths, with the fastest increase in incidence rate at 2%-3% annually from 2007 to 2016. In addition, the 5-year survival rate of liver cancer patients was only 18% during the follow-up from 2009 to 2015 [1]. The latest research believes that liver cancer almost develops in patients with chronic liver disease, driven by a vicious cycle of liver injury, inflammation, and regeneration that typically spans decades [2]. Liver cancer is identified to be highly heterogeneous, with poor prognosis and low response efficiency to therapeutic drugs [3, 4]. Despite recent advances that have been made in disease diagnosis and treatment, the long-term prognosis of liver cancer patients is still poor. Besides, the molecular mechanisms underlying recurrence and metastasis of liver cancer remain elusive. Therefore, it is of great significance to further explore accurate biomarkers and more effective therapeutic targets for liver cancer.

Regulator of calcineurin 1 (RCAN1) is an endogenous protein that interacts with calcineurin (CaN) and then interferes with the CaN-nuclear factor of activated T cells (NFAT) to inhibit its function [5]. RCAN1 locates on chromosome 21q22.12, and the overexpression of RCAN1 can induce neuronal apoptosis [6]. Recent studies displayed that RCAN1 hinders the proliferation and angiogenesis of endothelial cells [7], which regulates the malignant progression of most human cancers. A large dozens of excellent reviews describe that RCAN1 significantly hampers cell proliferation, migration, and invasion of liver cancer [8–10]. However, the regulatory mechanism targeting this gene is rarely reported. Accordingly, probing into the regulatory mechanism of RCAN1 in liver cancer may provide theoretical support for RCAN1 as a cancer regulator.

MicroRNAs (miRNAs) are small, noncoding RNA molecules that act a pivotal part in regulating gene expression by mediating their target genes. Studies provided some evidence that miRNAs are frequently dysregulated in tumor tissue and involved in multiple tumor-related processes, such as cell proliferation, apoptosis, migration, and invasion [11, 12]. Recent studies revealed that miRNAs affect the proliferation, migration, invasion, and apoptosis of liver cancer cells. For instance, miR-222 hastens cell proliferation and migration of liver cancer through BBC3; miR-498 restrains the invasion and migration of liver cancer through ZEB2 [13, 14]. Besides, numerous studies displayed that miRNAs affect the drug resistance of liver cancer tissue and cells. For example, miR-93 facilitates the sorafenib resistance of liver cancer by affecting PTEN/CDKN1A [15, 16]. Hence, miRNAs are considered as promising new targets for the treatment of liver cancer [17]. miR-182-5p, a member of the miR-183/96/182 cluster, appears as a miRNA of high-priority in liver cancer and related to varying cancers by serving as an oncogene or a tumor suppressor gene. For instance, miR-182-5p is identified as an oncogene in ovarian cancer [18], breast cancer [19], and melanoma [20], but a tumor suppressor in renal cell carcinoma [21, 22] and glioblastoma [23, 24]. Additionally, upregulation of miR-182-5p is proven to have diagnostic and prognostic value for liver cancer patients [25]. Although the role of miR-182-5p in tumor progression has been extensively studied, the research into the molecular mechanism of miR-182-5p in the occurrence and progression of liver cancer remains insufficient and is in need of further investigation.

In this study, we discovered through bioinformatics analysis that RCAN1 was differentially downregulated in liver cancer tissue, and there was a relationship between RCAN1 dysregulation and poor prognosis of patients with liver cancer. Our experimental results also revealed that miR-182-5p could downregulate the expression of RCAN1, thereby facilitating the progression of liver cancer.

## 2. Materials and Methods

### 2.1. Bioinformatics Analysis

Expression profiles of mature miRNAs (50 normal samples, 375 cancer samples) and mRNAs (50 normal samples, 374 cancer samples), along with relevant clinical data, were downloaded from the TCGA-LIHC dataset (https://portal.gdc.cancer.gov/). The expression of RCAN1 in normal tissue and cancer tissue was detected based on the downloaded data, with *t*-test used for verification. Patients were divided into high and low expression groups according to the median expression level of RCAN1 in all tumor samples, and survival analysis was then performed using the “survival” package. At the same time, a one-way analysis of variance (ANOVA) was applied to analyze the correlation between RCAN1 expression and clinical stage of tumor samples. The “EdgeR” package was employed for differential analysis to obtain differentially expressed miRNAs (DEmiRNAs) (∣logFC | >2.0, *p*adj < 0.01). To clarify the upstream regulator miRNA of RCAN1 in liver cancer, mirDIP (http://ophid.utoronto.ca/mirDIP/index.jsp#r) and starBase (http://starbase.sysu.edu.cn/) databases were consulted. Based on the competing endogenous RNA (ceRNA) hypothesis that miRNA is negatively correlated with their target mRNA, the predicted miRNAs were intersected with the upregulated DEmiRNAs. Pearson correlation analysis was carried out for the obtained miRNAs and RCAN1 to gain the research target miRNA with the strongest negative correlation with RCAN1. Then, the expression of the miRNA in TCGA samples was further clarified with *t*-test performed to identify statistical significance.

### 2.2. Cell Culture

The following cell lines were adopted for this study: human normal liver cell line HL-7702 [L-02] (BNCC100012), liver cancer cell lines SMMC-7721 (BNCC338089), HepG2 (BNCC338070), Huh-7 (BNCC337690), and Hep3b (BNCC337952). All the cell lines were purchased from BeNa Culture Collection (BNCC, China). SMMC-7721, HepG2, Huh-7, and Hep3b cell lines were all cultured in Dulbecco's modified Eagle medium (DMEM) (BNCC351841, BNCC, China) with 10% fetal bovine serum (FBS), while HL-7702 [L-02] cell line was cultivated in RPMI-1640 medium (BNCC341471, BNCC, China) supplemented with 10% FBS. All the cell lines were maintained in an incubator at 37°C with 5% CO_2_.

### 2.3. Cell Transfection

RCAN1 overexpressed vector pcDNA3.1-RCAN1 (oe-RCAN1), miR-182-5p mimic (miR-mimic), negative control vector pcDNA3.1 (oe-NC), and control mimic (miR-NC) were all obtained from GenePharma (Shanghai, China). To study the effects of miR-182-5p and RCAN1 on cell proliferation, migration, and invasion, Hep3b cells were cultured in a corresponding culture medium for at least 24 h and washed with phosphate-buffered saline (PBS, pH 7.4). Then, the cells were subjected to transfection using Lipofectamine 2000 (Invitrogen) according to the manufacturer's protocol and sequentially cultured in the corresponding medium at 37°C with 5% CO_2_.

### 2.4. Real-Time Quantitative Polymerase Chain Reaction (qRT-PCR)

Total RNA was isolated from cells using TRIzol reagent (Invitrogen) following the manufacturer's instructions. The RNA extracted from each group was quantified by the NanoDrop spectrophotometer (NanoDrop, Wilmington, DE, USA), and then complementary DNA (cDNA) was synthesized by the reverse transcription kit (Applied Biosystems, Foster City, CA, USA). The synthesized cDNA was diluted with the SYBR Green Real-time PCR Master Mix (Toyobo, Tokyo, Japan) per the amplification instructions. Next, qRT-PCR was run on the Applied Biosystems ® 7500 Real-Time PCR System (Thermo Fisher Scientific, Waltham, MA) to measure the expression of miR-182-5p and RCAN1 mRNA, with U6 and *β*-actin applied as internal references, respectively. The relative expression of miR-182-5p and RCAN1 mRNA between the control group and the experimental group was analyzed via the 2^-*ΔΔ*Ct^ value. The experiment was carried out in triplicate and repeated three times. The primer sequences are detailed in Table 1.

### 2.5. Cell Counting Kit-8 (CCK-8) Assay

CCK-8 analysis was performed to evaluate cell proliferation of the liver cancer cell line Hep3b. The cells were seeded into a 96-well plate (1 × 10^4^ cells/well) overnight. Following that, CCK-8 reagent (Sigma, St. Louis, MO, USA) was added to each well at 0, 24, 48, 72, and 96 h after transfection, followed by 2 h of cell incubation at 37°C. The optical density (OD) at 450 nm was measured with the enzyme-labeled instrument (Bio-Rad, Hercules, CA, USA).

### 2.6. Wound Healing Assay

First, the liver cancer cells Hep3b (5 × 10^5^) were inoculated into a 6-well plate overnight. Then, the cell monolayer was scraped with a 100 *μ*L sterile pipette tip, followed by an addition of a serum-free medium for further culture. Next, wounds were photographed with a microscope, and cell migration was observed at 0 and 24 h. At last, the wound healing percentage was calculated using ImageJ.

### 2.7. Transwell Invasion Assay

Cell invasion was analyzed through Transwell assay. Hep3b cells (1 × 10^5^ cells/mL) suspended by serum-free medium were placed in the upper chamber (Costar, Corning, NY, USA) precoated with Matrigel (BD Biosciences, San Jose, CA, USA), while serum-supplemented medium was added into the lower chamber. After 24 h of culture at 37°C, the cells that did not pass through the membrane were softly wiped off with a cotton swab. The cells on the basal side of the membrane were fixed with 4% paraformaldehyde for 10 min, stained with crystal violet (Sigma), and then counted under the microscope (Olympus, Tokyo, Japan). Three fields were randomly selected, and the average number of cells in each field was calculated as well.

### 2.8. Western Blot

According to the manufacturer's instructions, the total proteins were extracted using radio-immunoprecipitation analysis (RIPA) lysis buffer (Beyotime, Shanghai, China). After separation by 10% sodium dodecyl sulfate-polyacrylamide gel electrophoresis (SDS-PAGE), the proteins were transferred onto a polyvinylidene fluoride (PVDF) membrane. The membrane was incubated overnight at 4°C with the following primary antibodies: rabbit anti-RCAN1 (Abcam, UK) and rabbit anti-*β*-actin (Abcam, UK). After the incubation with primary antibodies, the membrane was washed with 0.1% TBST for 3 times, 10 min for each time. The membrane was incubated at room temperature for 2 h with secondary antibody goat anti-rabbit IgG H&L (HRP) (Abcam, UK) and washed with PBST buffer 3 times, 10 min for each time. Protein expression was detected using an enhanced chemiluminescence kit (GE Healthcare, Chicago, IL, USA) with *β*-actin as an internal reference.

### 2.9. Dual-Luciferase Reporter Gene Assay

The wild-type (WT) RCAN1 3′-UTR sequence was amplified and cloned into the pmirGLO reporter (Promega) to generate the RCAN1-WT plasmid vector. The TaKaRa MutanBEST kit (TaKaRa) was handled to construct mutant (MUT) RCAN1 3′-UTR plasmid vector (RCAN1-MUT). Then, the RCAN1-WT/MUT reporter vector and miR-mimic/miR-NC were cotransfected into Hep3b cells with Lipofectamine 2000 (Thermo Fisher Scientific). After 48 h of transfection, luciferase activities of cells were assessed by the luciferase reporter gene system (Promega).

### 2.10. Flow Cytometry

Liver cancer cells Hep3b in each group were collected, treated with trypsin, and fixed at 4°C in 7% ethanol overnight. After being washed 3 times in cold PBS, the cells were resuspended in 1 mL PBS containing 40 *μ*g PI and 100 *μ*g RNase A (Sigma-Aldrich, St Louis, MO) at 37°C for 30 min of incubation. The samples were then analyzed using the BD FACSAria III cell sorter (San Jose, CA, USA).

### 2.11. Statistical Analysis

All data were collected from at least two or three independent experiments. GraphPad Prism 7 was employed for statistical analysis. All measurement data were presented as mean ± SD, while the statistical significance of the difference between the two groups was determined by *t*-test. *p* < 0.05 was considered statistically significant.

## 3. Results

### 3.1. RCAN1 Is Notably Lowly Expressed in Liver Cancer Cells

**Figure 1:**
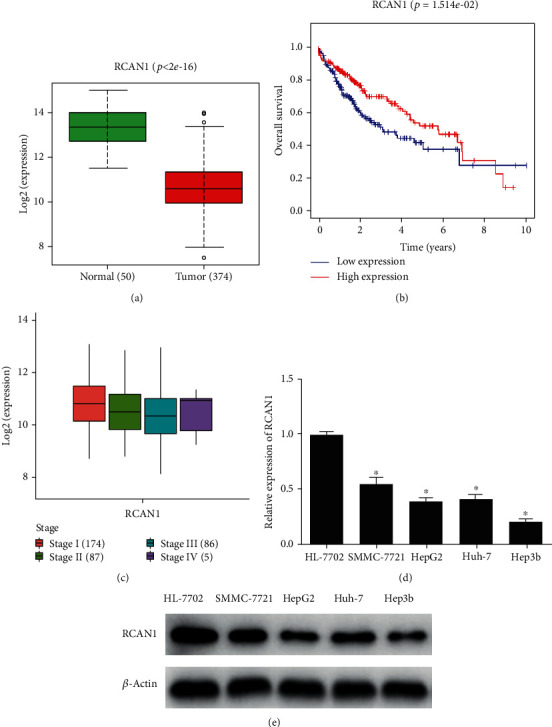
RCAN1 is significantly lowly expressed in liver cancer cells. (a) Relative expression of RCAN1 in normal group and tumor group in TCGA (the green represents normal group, the red represents tumor group); (b) survival curves of patients based on the expression of RCAN1 (the red line represents high expression group, the blue line represents low expression group); (c) correlation analysis of RCAN1 expression and clinical stage; (d) the expression levels of RCAN1 in human normal liver cell line HL-7702 and liver cancer cell lines SMMC-7721, HepG2, Huh-7, and Hep3b were detected by qRT-PCR; (e) RCAN1 protein levels in human normal liver cell line HL-7702 and liver cancer cell lines SMMC-7721, HepG2, Huh-7, and Hep3b were detected by western blot; ^∗^*p* < 0.05.

As stated by the gene expression data in the TCGA-LIHC dataset, RCAN1 exhibited highly decreased expression in liver cancer tissue (Figure 1(a)). The survival analysis demonstrated that its low expression indicated poor prognosis (Figure 1(b)). Besides, RCAN1 had a notable correlation with the clinical stage of liver cancer (Figure 1(c)). Next, the expression of RCAN1 in human normal liver cell line HL-7702 and four liver cancer cell lines SMMC-7721, HepG2, Huh-7, and Hep3b was assessed via qRT-PCR. It could be found that the expression of RCAN1 mRNA in liver cancer cell lines was dramatically decreased (Figure 1(d)). Then, the results of western blot denoted that the expression of RCAN1 protein in liver cancer cell lines was prominently downregulated as well (Figure 1(e)). The above results displayed that RCAN1 was remarkably lowly expressed in liver cancer cells. To better explore the mechanism of RCAN1 in liver cancer cells, the Hep3b cell line with the lowest expression of RCAN1 was selected for follow-up experiments.

### 3.2. Overexpression of RCAN1 Hampers the Progression of Liver Cancer Cells

**Figure 2:**
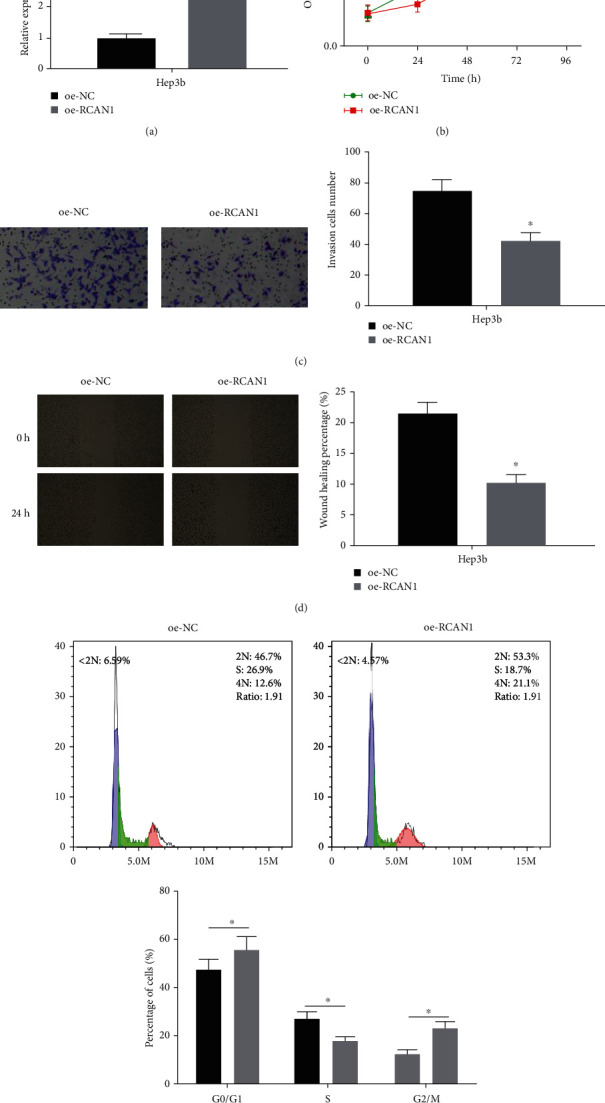
Overexpression of RCAN1 inhibits the progression of liver cancer cells. (a) The transfection efficiency of RCAN1 in liver cancer cells Hep3b was detected by qRT-PCR; (b) the proliferative ability of Hep3b cells in different transfection groups was assessed through CCK-8 assay; (c) the invasive ability of Hep3b cells in different transfection groups was detected by Transwell assay (100×); (d) the migratory ability of Hep3b cells in different transfection groups was measured via wound healing assay (40×); (e) changes of Hep3b cell cycle in different transfection groups were assessed through flow cytometry; ^∗^*p* < 0.05.

RCAN1 overexpressed Hep3b cells were established to study the role of RCAN1 in liver cancer cells. The transfection efficiency in different treatment groups was measured through qRT-PCR. The results revealed that the expression of RCAN1 in the oe-RCAN1 group was outstandingly upregulated (Figure 2(a)), showing that the constructed cells could be taken for subsequent experiments. Then, it was illustrated through CCK-8 assay that overexpression of RCAN1 conspicuously suppressed Hep3b proliferation (Figure 2(b)). Next, the Transwell assay was practiced to detect whether RCAN1 regulates the invasion of liver cancer cells, and it came upon that the invasive ability of cells was strikingly reduced with overexpression of RCAN1 (Figure 2(c)). Besides, the inhibitory effect of overexpressed RCAN1 on the migratory ability of liver cancer cells was also revealed by wound healing assay (Figure 2(d)). Moreover, in view of the cell cycle analysis through flow cytometry, RCAN1 might impede the G1-S transition in the cells, thereby suppressing the cells from undergoing mitosis. Thus, it could be seen that the overexpression of RCAN1 pronouncedly hampered the progression of the Hep3b cell cycle (Figure 2(e)). These results signified that overexpression of RCAN1 repressed cell proliferation, migration, invasion, and cell cycle progression of liver cancer.

### 3.3. mir-182-5p Is Highly Expressed in Liver Cancer Cells

**Figure 3:**
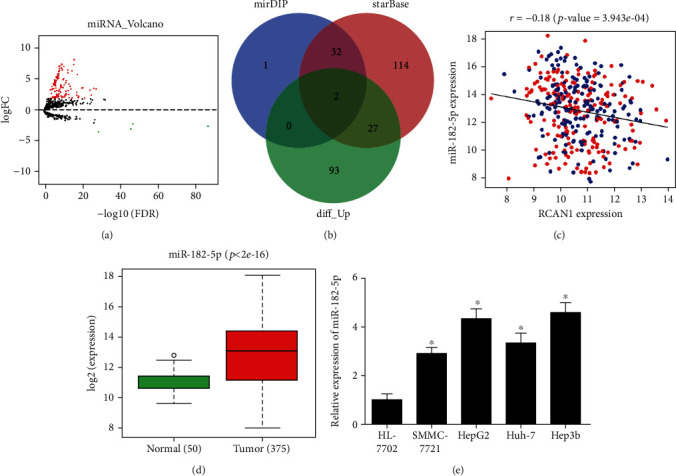
miR-182-5p is highly expressed in liver cancer cells. (a) Volcano map of DEmiRNAs in liver cancer data set of TCGA database (red represents up-regulated genes, green represents downregulated genes); (b) Venn diagram of predicted upstream miRNAs of RCAN1 and DEmiRNAs; (c) Pearson correlation analysis between RCAN1 and miR-182-5p; (d) Relative expression of miR-182-5p in the normal group and tumor group in TCGA (green represents the normal group, red represents the tumor group); (e) the expression of miR-182-5p in the human normal liver cell line HL -7702 and human liver cancer cell lines SMMC-7721, HepG2, Huh-7, and Hep3b was detected by qRT-PCR; ^∗^*p* < 0.05.

In order to investigate the upstream regulatory mechanism of RCAN1 in liver cancer cells, 126 DEmiRNAs containing 122 upregulated and 4 downregulated genes were firstly obtained through bioinformatics analysis in TCGA (Figure 3(a)). Then, upstream miRNAs of RCAN1 were predicted through the mirDIP and starBase databases. The predicted miRNAs were intersected with the 122 upregulated DEmiRNAs to obtain 2 miRNAs that have binding sites with RCAN1 (Figure 3(b)). The Pearson correlation analysis exhibited that RCAN1 and miR-182-5p were inversely correlated with higher Pearson correlation coefficient (Figure 3(c)). As revealed by TCGA data, compared with normal tissue, liver cancer tissue had notably elevated expression of miR-182-5p (Figure 3(d)). Thus, this study took miR-182-5p as the research object. At last, the expression of miR-182-5p in the four liver cancer cell lines and the human normal liver cell line HL-7702 was measured by qRT-PCR. The results also clarified that the expression of miR-182-5p in liver cancer cell lines was significantly upregulated (Figure 3(e)). The above results suggested that miR-182-5p was highly expressed in liver cancer cells, and it was likely to act as an oncogene in the progression of liver cancer cells.

### 3.4. mir-182-5p Targets RCAN1 in Liver Cancer Cells

**Figure 4:**
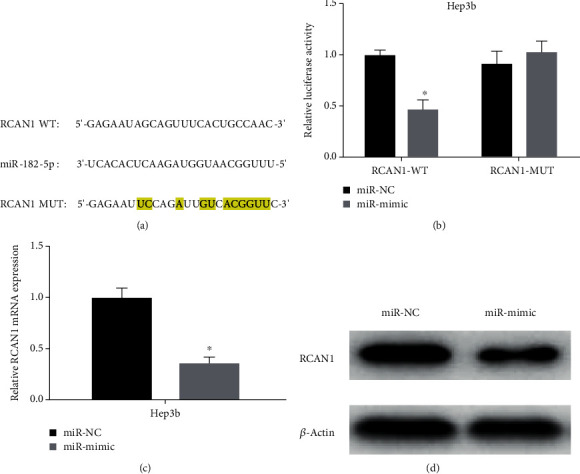
miR-182-5p targets RCAN1 in liver cancer cells. (a) The targeted binding sites of miR-182-5p and RCAN1 were predicted via bioinformatics; (b) The luciferase activities of the Hep3b cell line in different transfection groups were measured by dual-luciferase reporter gene assay; (c) the expression of RCAN1 mRNA in different transfection groups of the Hep3b cell line was assessed through qRT-PCR; (d) the protein expression of RCAN1 in Hep3b cell line in various transfection groups was detected by western blot; ^∗^*p* < 0.05.

To further validate the molecular regulation of miR-182-5p on RCAN1, the targeted binding sites of miR-182-5p on RCAN1 3′UTR were predicted and uncovered via bioinformatics (Figure 4(a)). Then, the binding relationship between miR-182-5p and RCAN1 was verified via dual-luciferase reporter gene assay. It illustrated that overexpression of miR-182-5p inhibited the luciferase activity of cells with RCAN1-WT, while there was no influence on the luciferase activity of cells with RCAN1-MUT (Figure 4(b)). Then, the expression of RCAN1 mRNA in different transfection groups of the Hep3b cell line was assessed through qRT-PCR. The results exhibited that it was dramatically decreased in the miR-182-5p overexpressed cells (Figure 4(c)). Western blot also denoted that protein expression of RCAN1 decreased prominently with overexpression of miR-182-5p (Figure 4(d)). The above results presented that miR-182-5p hindered the expression of RCAN1 in liver cancer cells by targeting RCAN1.

### 3.5. mir-182-5p Promotes Cell Progression of Liver Cancer via Targeting RCAN1

**Figure 5:**
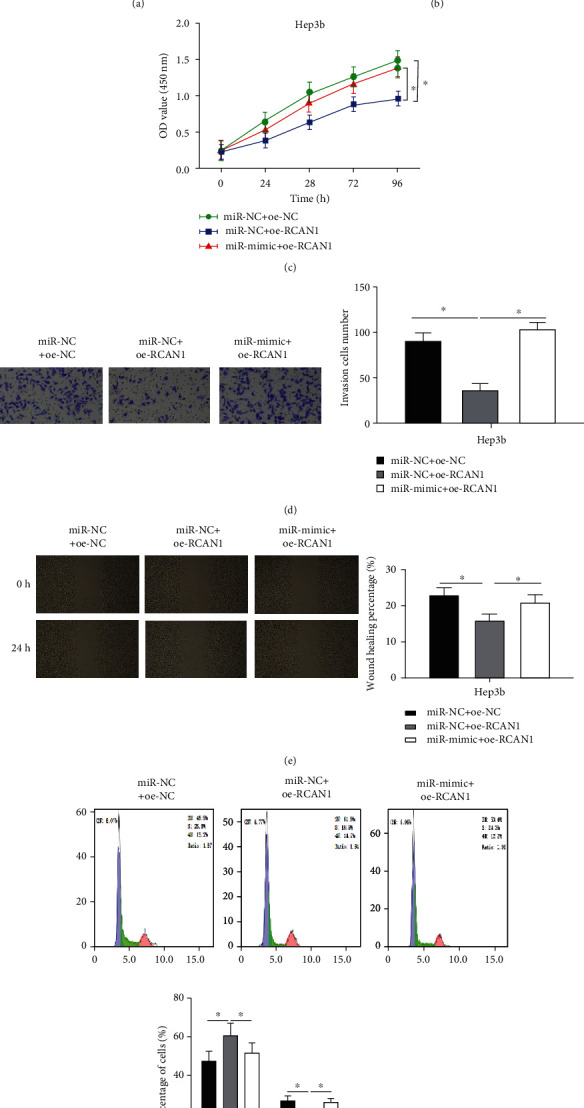
miR-182-5p promotes cell progression of liver cancer via targeting RCAN1. (a) The expression levels of RCAN1 mRNA in Hep3b cells in different transfection groups (miR − NC + oe − NC, miR − NC + oe − RCAN1, and miR − mimic + oe − RCAN1) were detected by qRT-PCR; (b) the protein expression of RCAN1 in Hep3b cells in different transfection groups was assessed through western blot; (c) the proliferative ability of Hep3b cells in different transfection groups was measured via CCK-8 assay; (d) the invasive ability of Hep3b cells in different transfection groups was measured by Transwell assay (100×); (e) the migratory ability of Hep3b cells in different transfection groups was assessed via wound healing assay (40×); (f) changes of Hep3b cell cycle in different transfection groups were tested by flow cytometry; ^∗^*p* < 0.05.

To explore whether miR-182-5p participates in the regulation of liver cancer cell function by targeting RCAN1, cell lines with overexpressed RCAN1 (miR − NC + oe − RCAN1) or cell lines with simultaneously overexpressed miR-182-5p and RCAN1 (miR − mimic + oe − RCAN1) were constructed. First, the expression of RCAN1 in each group was observed by qRT-PCR and western blot. It was disclosed that the mRNA and protein expression of RCAN1 in the miR − NC + oe − RCAN1 group increased significantly, while then decreased in the miR − mimic + oe − RCAN1 group (Figures 5(a) and 5(b)). CCK-8 assay manifested that overexpression of RCAN1 remarkably lessened the proliferative ability of liver cancer cells, while overexpression of miR-182-5p and RCAN1 simultaneously diminished the inhibitory effect of RCAN1 overexpression on cell proliferation (Figure 5(c)). Next, the migration and invasion of liver cancer cells in each transfection group were further tested by wound healing assay and Transwell assay. It came upon that the migratory and invasive abilities of cancer cells were outstandingly reduced with overexpression of RCAN1, whereas the inhibitory effect was notably restored when miR-182-5p and RCAN1 were overexpressed concurrently (Figures 5(d) and 5(e)). Besides, flow cytometry also confirmed that RCAN1 prevented cells from undergoing mitosis by blocking the transition of cells in the G1 phase to the S phase, which conspicuously restrained the cell cycle progression of the Hep3b cells, while the repressed progression was rescued with overexpressed miR-182-5p and RCAN1 (Figure 5(f)). In summary, our study conveyed that miR-182-5p fostered cell proliferation, migration, invasion, and cell cycle progression of liver cancer by targeting RCAN1 expression.

## 4. Discussion

Liver cancer is the fifth most common cancer in the world with mortality on the rise [26]. The main challenges of liver cancer treatment include intrahepatic recurrence and metastasis, which lead to a dismal prognosis [27]. Previous studies attested that miRNAs play a key role in the occurrence and progression of liver cancer cells. For instance, miR-498 [14], miR-34a [28], miR-26b [29], miR-196a [30], and other miRNAs are pronouncedly lowly expressed in liver cancer cells and can be used as biomarkers. Therefore, we explored therapeutic methods at the molecular level to find potential biomarkers and therapeutic targets.

Initially, we learned from the data retrieved from the bioinformatics database that RCAN1 was particularly lowly expressed in liver cancer tissue. qRT-PCR and western blot revealed that the mRNA and protein expression levels of RCAN1 in liver cancer cells were notably lower than those in normal human liver cells, indicating that RCAN1 may participate in regulating the progression of liver cancer. Further functional experiments exhibited that overexpression of RCAN1 suppressed cell proliferation, migration, invasion, and cell cycle progression of liver cancer. Thus, RCAN1 served as a tumor suppressor to regulate the occurrence and progression of liver cancer. Previously, it was documented that RCAN1 acts as a tumor suppressor in various cancers. For instance, RCAN1 is appreciably lowly expressed in oral squamous cell carcinoma, while miR-103a-3p directly targets RCAN1 to boost cell proliferation, migration, and invasion [31]; RCAN1 in hepatocellular carcinoma (HCC) is downregulated by circADAMTS14 and miR-572 to mediate the progression of cancer cells [32]. This study further clarified that RCAN1 could act as a tumor suppressor in liver cancer cells to hamper cell occurrence and progression.

miR-182-5p as a gene with complicated functions can serve as an oncogene or a tumor suppressor gene in different cancers. Studies illustrated that miR-182-5p stimulates the proliferation of renal cell carcinoma by activating the AKT/FOXO3a signaling pathway [21]; miR-182-5p hastens the growth of oral squamous cell carcinoma through repressing CAMK2N1 [33]; miR-182-5p eliminates cell proliferation and migration of ovarian cancer via targeting BNIP3 [34]; miR-182-5p alleviates the proliferation and metastasis of colorectal cancer by targeting MTDH [35]. Wang et al. [36] declared that miR-182-5p fosters HCC metastasis by targeting metastasis suppressor 1 (MTSS1). In this study, we disclosed that miR-182-5p was markedly highly expressed in liver cancer tissue and cell lines, via bioinformatics analysis and qRT-PCR. Additionally, the dual-luciferase reporter gene assay further proved the targeted binding relationship between miR-182-5p and RCAN1, while qRT-PCR and western blot also confirmed that RCAN1 mRNA and protein were negatively regulated by miR-182-5p. Rescue experiments identified that overexpression of RCAN1 conspicuously hindered cell proliferation, migration, invasion, and cell cycle progression of liver cancer, while overexpressing miR-182-5p and RCAN1 simultaneously weakened such inhibitory effect. These results deepened our understanding of the regulatory mechanism of miR-182-5p in liver cancer cells. In the treatment of liver cancer, the abnormal expression of miR-182-5p is the potential to be a valuable biomarker and candidate indicator.

This study, to sum up, confirmed that the relationship between miR-182-5p and RCAN1 could affect the progression of liver cancer cells. However, this study is still subject to certain limitations. For instance, this study only substantiated the effects of RCAN1 and miR-182-5p on the proliferation, migration, and invasion of liver cancer cells through *in vitro* cell experiments, whereas the prognostic value of miR-182-5p for liver cancer patients is not fully verified through clinical studies. In fact, numerous studies manifested that miRNAs can affect the prognosis of patients in various forms like directly affecting the metastasis and invasion of cancer or through affecting drug resistance [37, 38]. There was a study indicating that patients with high miR-182-5p expression in varying cancers, such as breast cancer [39] and pancreatic cancer [40], have shorter survival times. This study speculated that the expression of miR-182-5p possesses an inverse association with the prognosis of patients with liver cancer. However, further clinical experiments need to be carried out to substantiate the prognostic value of miR-182-5p in patients with liver cancer. In the future, our team will further investigate the application value of miR-182-5p in the prognostic prediction of liver cancer.

Viewed in toto, these findings suggested that miR-182-5p/RCAN1 may have diagnostic potential in liver cancer, and RCAN1 was regulated by its upstream regulator miR-182-5p to act as a tumor suppressor. This study revealed, for the first time, the regulatory mechanism of the interaction between miR-182-5p and RCAN1 underlying the progression of liver cancer, which may provide superior treatment opportunities.

## Figures and Tables

**Table 1:** Primer sequences in qRT-PCR.

Target gene	Gene sequences
miR-182-5p	F: 5′-TGCGGTTTG GCAATGGTAGAAC-3′
	R: 5′-CCAGTGCAGGGTCCGAGGT-3′
U6	F: 5′-CTCGCTTCGGCAGCACA-3′
	R: 5′-AACGCTTCACGAATTTGCGT-3′
RCAN1	F: 5′-AGGACGTATGACAAGGACAT-3′
	R: 5′-ATCAGAAACTGCTTGTCTGGA-3′
*β*-Actin	F: 5′-CAGCCTTCCTTCTTGGGTAT-3′
	R: 5′-TGGCATAGAGGTCTTTACGG-3′

## Data Availability

No data were used to support this study.

## References

[1] Siegel R. L., Miller K. D., Jemal A. (2020). Cancer statistics, 2020. *CA: a Cancer Journal for Clinicians*.

[2] Yu L. X., Schwabe R. F. (2017). The gut microbiome and liver cancer: mechanisms and clinical translation. *Nature Reviews. Gastroenterology & Hepatology*.

[3] Qiu Z., Li H., Zhang Z. (2019). A pharmacogenomic landscape in human liver cancers. *Cancer Cell*.

[4] Kudo M., Finn R. S., Qin S. (2018). Lenvatinib versus sorafenib in first-line treatment of patients with unresectable hepatocellular carcinoma: a randomised phase 3 non-inferiority trial. *Lancet*.

[5] Chan B., Greenan G., McKeon F., Ellenberger T. (2005). Identification of a peptide fragment of DSCR1 that competitively inhibits calcineurin activity in vitro and in vivo. *Proceedings of the National Academy of Sciences of the United States of America*.

[6] Sun X., Wu Y., Chen B. (2011). Regulator of calcineurin 1 (RCAN1) facilitates neuronal apoptosis through caspase-3 activation. *The Journal of Biological Chemistry*.

[7] Minami T., Miura M., Aird W. C., Kodama T. (2006). Thrombin-induced autoinhibitory factor, Down syndrome critical region-1, attenuates NFAT-dependent vascular cell adhesion molecule-1 expression and inflammation in the endothelium. *The Journal of Biological Chemistry*.

[8] Jin H., Wang C., Jin G. (2017). Regulator of calcineurin 1 gene isoform 4, down-regulated in hepatocellular carcinoma, prevents proliferation, migration, and invasive activity of cancer cells and metastasis of orthotopic tumors by inhibiting nuclear translocation of NFAT1. *Gastroenterology*.

[9] Wang C., Saji M., Justiniano S. E. (2017). RCAN1-4 is a thyroid cancer growth and metastasis suppressor. *JCI Insight*.

[10] Shi B., Zhang X., Chao L. (2018). Comprehensive analysis of key genes, microRNAs and long non-coding RNAs in hepatocellular carcinoma. *FEBS Open Bio*.

[11] Fendler A., Jung K. (2013). MicroRNAs as new diagnostic and prognostic biomarkers in urological tumors. *Critical Reviews in Oncogenesis*.

[12] Fendler A., Stephan C., Yousef G. M., Jung K. (2011). MicroRNAs as regulators of signal transduction in urological tumors. *Clinical Chemistry*.

[13] Liu Z., Sun J., Liu B., Zhao M., Xing E., Dang C. (2018). miRNA‑222 promotes liver cancer cell proliferation, migration and invasion and inhibits apoptosis by targeting BBC3. *International Journal of Molecular Medicine*.

[14] Zhang X., Xu X., Ge G. (2018). miR498 inhibits the growth and metastasis of liver cancer by targeting ZEB2. *Oncology Reports*.

[15] Wei L., Wang X., Lv L. (2019). The emerging role of microRNAs and long noncoding RNAs in drug resistance of hepatocellular carcinoma. *Molecular Cancer*.

[16] Ohta K., Hoshino H., Wang J. (2015). MicroRNA-93 activates c-Met/PI3K/Akt pathway activity in hepatocellular carcinoma by directly inhibiting PTEN and CDKN1A. *Oncotarget*.

[17] Gnoni A., Santini D., Scartozzi M. (2015). Hepatocellular carcinoma treatment over sorafenib: epigenetics, microRNAs and microenvironment. Is there a light at the end of the tunnel?. *Expert Opinion on Therapeutic Targets*.

[18] Xu X., Ayub B., Liu Z. (2014). Anti-miR182 reduces ovarian cancer burden, invasion, and metastasis: an in vivo study in orthotopic xenografts of nude mice. *Molecular Cancer Therapeutics*.

[19] Li P., Sheng C., Huang L. (2014). MiR-183/-96/-182 cluster is up-regulated in most breast cancers and increases cell proliferation and migration. *Breast Cancer Research*.

[20] Segura M. F., Hanniford D., Menendez S. (2009). Aberrant miR-182 expression promotes melanoma metastasis by repressing FOXO3 and microphthalmia-associated transcription factor. *Proceedings of the National Academy of Sciences of the United States of America*.

[21] Xu X., Wu J., Li S. (2014). Downregulation of microRNA-182-5p contributes to renal cell carcinoma proliferation via activating the AKT/FOXO3a signaling pathway. *Molecular Cancer*.

[22] Wang X., Li H., Cui L., Feng J., Fan Q. (2016). MicroRNA-182 suppresses clear cell renal cell carcinoma migration and invasion by targeting IGF1R. *Neoplasma*.

[23] Kouri F. M., Ritner C., Stegh A. H. (2015). miRNA-182 and the regulation of the glioblastoma phenotype - toward miRNA-based precision therapeutics. *Cell Cycle*.

[24] Kouri F. M., Hurley L. A., Daniel W. L. (2015). miR-182 integrates apoptosis, growth, and differentiation programs in glioblastoma. *Genes & Development*.

[25] Chen L., Chu F., Cao Y., Shao J., Wang F. (2015). Serum miR-182 and miR-331-3p as diagnostic and prognostic markers in patients with hepatocellular carcinoma. *Tumour Biology*.

[26] Negoita S., Feuer E. J., Mariotto A. (2018). Annual report to the nation on the status of cancer, part II: recent changes in prostate cancer trends and disease characteristics. *Cancer*.

[27] Singal A. G., El-Serag H. B. (2015). Hepatocellular carcinoma from epidemiology to prevention: translating knowledge into practice. *Clinical Gastroenterology and Hepatology*.

[28] Zhang H. F., Wang Y. C., Han Y. D. (2018). MicroRNA‑34a inhibits liver cancer cell growth by reprogramming glucose metabolism. *Molecular Medicine Reports*.

[29] Feng Y., Zu L. L., Zhang L. (2018). MicroRNA-26b inhibits the tumor growth of human liver cancer through the PI3K/Akt and NF-*κ*B/MMP-9/VEGF pathways. *Oncology Reports*.

[30] Yang L., Peng F., Qin J., Zhou H., Wang B. (2017). Downregulation of microRNA-196a inhibits human liver cancer cell proliferation and invasion by targeting FOXO1. *Oncology Reports*.

[31] ZHANG G., CHEN Z., ZHANG Y., LI T., BAO Y., ZHANG S. (2020). Inhibition of miR-103a-3p suppresses the proliferation in oral squamous cell carcinoma cells via targeting RCAN1. *Neoplasma*.

[32] Song C., Li D., Liu H. (2018). The competing endogenous circular RNA ADAMTS14 suppressed hepatocellular carcinoma progression through regulating microRNA-572/regulator of calcineurin 1. *Journal of Cellular Physiology*.

[33] Li N., Nan C. C., Zhong X. Y. (2018). miR-182-5p promotes growth in oral squamous cell carcinoma by inhibiting CAMK2N1. *Cellular Physiology and Biochemistry*.

[34] Jia X. N., Yin S. D., Wei Y., Chen L. (2019). MiR-182-5p inhibited proliferation and migration of ovarian cancer cells by targeting BNIP3. *European Review for Medical and Pharmacological Sciences*.

[35] Jin Y., Zhang Z. L., Huang Y., Zhang K. N., Xiong B. (2019). MiR-182-5p inhibited proliferation and metastasis of colorectal cancer by targeting MTDH. *European Review for Medical and Pharmacological Sciences*.

[36] Wang J., Li J., Shen J., Wang C., Yang L., Zhang X. (2012). MicroRNA-182 downregulates metastasis suppressor 1 and contributes to metastasis of hepatocellular carcinoma. *BMC Cancer*.

[37] Brunetti O., Gnoni A., Licchetta A. (2019). Predictive and prognostic factors in HCC patients treated with sorafenib. *Medicina (Kaunas)*.

[38] Cao M. Q., You A. B., Zhu X. D. (2018). Correction to: miR-182-5p promotes hepatocellular carcinoma progression by repressing FOXO3a. *Journal of Hematology & Oncology*.

[39] Zhao Y. S., Yang W. C., Xin H. W., Han J. X., Ma S. G. (2019). MiR-182-5p knockdown targeting PTEN inhibits cell proliferation and invasion of breast cancer cells. *Yonsei Medical Journal*.

[40] Li C., du X., Tai S. (2014). GPC1 regulated by miR-96-5p, rather than miR-182-5p, in inhibition of pancreatic carcinoma cell proliferation. *International Journal of Molecular Sciences*.

